# Pan–ice-sheet glacier terminus change in East Antarctica reveals sensitivity of Wilkes Land to sea-ice changes

**DOI:** 10.1126/sciadv.1501350

**Published:** 2016-05-06

**Authors:** Bertie W. J. Miles, Chris R. Stokes, Stewart S. R. Jamieson

**Affiliations:** Department of Geography, Durham University, Science Site, South Road, Durham DH1 3LE, UK.

**Keywords:** East Antarctic Ice Sheet, Antarctica, sea ice, glaciers, Wilkes Land

## Abstract

The dynamics of ocean-terminating outlet glaciers are an important component of ice-sheet mass balance. Using satellite imagery for the past 40 years, we compile an approximately decadal record of outlet-glacier terminus position change around the entire East Antarctic Ice Sheet (EAIS) marine margin. We find that most outlet glaciers retreated during the period 1974–1990, before switching to advance in every drainage basin during the two most recent periods, 1990–2000 and 2000–2012. The only exception to this trend was in Wilkes Land, where the majority of glaciers (74%) retreated between 2000 and 2012. We hypothesize that this anomalous retreat is linked to a reduction in sea ice and associated impacts on ocean stratification, which increases the incursion of warm deep water toward glacier termini. Because Wilkes Land overlies a large marine basin, it raises the possibility of a future sea level contribution from this sector of East Antarctica.

## Teaser

Recent retreat of outlet glaciers in Wilkes Land, East Antarctica, is driven by changes in sea ice.

## INTRODUCTION

A growing body of evidence suggests that large marine basins in the East Antarctic Ice Sheet (EAIS) have made significant contributions to sea level during previous climatic periods when global CO_2_ concentrations were comparable to today’s values (*1*–*3*). Despite this, recent estimates suggest that the current mass balance of the EAIS has been in equilibrium or slightly positive over the past decade (*4*–*6*). However, there are clear regional variations, with mass gained in Dronning Maud Land and Enderby Land and a clear signal of mass loss in Wilkes Land (*4*–*6*). This is significant because Wilkes Land overlies the Aurora Subglacial Basin. Because of its reverse bed slope and deep troughs (*3*), this basin may have caused marine instability in the past (*1*), similar to that being proposed for West Antarctica (*7*, *8*).

**Fig. 1:**
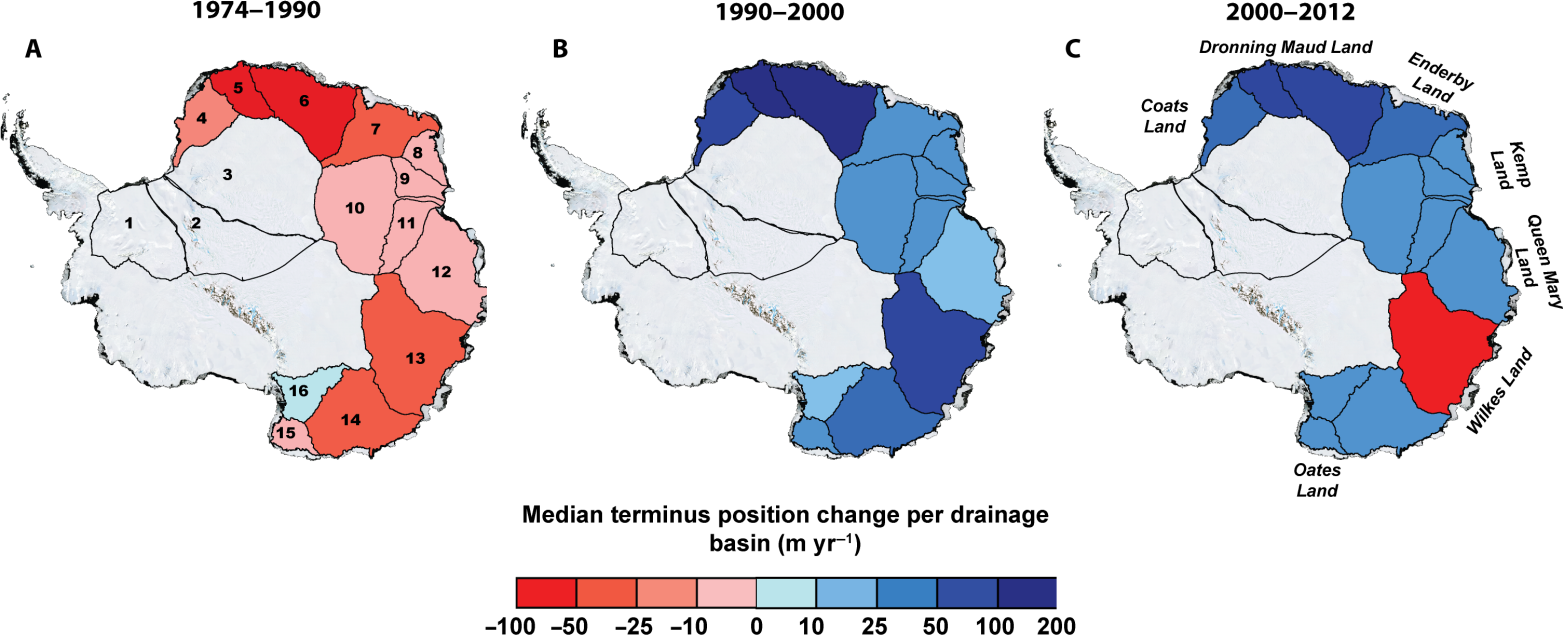
Median rate of East Antarctic outlet-glacier terminus position change in each drainage basin (red, retreat; blue, advance). (**A** to **C**) Data for (A) 1974–1990, (B) 1990–2000, and (C) 2000–2012. Note the anomalous retreat of outlet glaciers in Wilkes Land between 2000 and 2012. All glacier terminus position measurements are presented in database S1.

Critical to marine ice-sheet instability are ice shelves and the floating extension of outlet glaciers, which may act to buttress ice flow from the interior of the ice sheet (*9*). Recent modeling studies have suggested that changes in the extent of floating termini are an important control for the stability of the EAIS (*10*). However, despite recent reports of the heightened sensitivity of some East Antarctic outlet glaciers to changes in the ocean climate system (*11*), there has yet to be a comprehensive analysis of outlet-glacier terminus position changes around the whole EAIS marine margin in relation to changes in mass balance and potential oceanic or atmospheric forcings. We address this by compiling a pan–ice-sheet record of outlet-glacier terminus change over the past 40 years. To minimize the potential influence of short-term interannual variations and major (potentially stochastic) calving events, we focused on approximately decadal time steps (1974, 1990, 2000, and 2012), for which satellite image availability covered the entire marine margin (Materials and Methods). Terminus position changes, stretching from the Ronne Ice Shelf to Queen Mary Land, were mapped (Fig. 1 and fig. S1), adding 176 ocean-terminating outlet glaciers to the previously published record of Miles *et al*. (*11*). In total, 351 outlet glaciers, spanning the entire EAIS marine margin, are included in our estimates. To allow comparison to several recent studies reporting changes in mass balance (*4*–*6*), we analyzed terminus position change in each previously defined drainage basin (*12*).

## RESULTS AND DISCUSSION

Our results show clear decadal-scale patterns of terminus position around the entire EAIS marine margin (table S1). During the 1974–1990 epoch, 65% of glaciers retreated at a median rate of 17.8 m year^−1^. From 1990 to 2000, however, there was a highly significant (*P* < 0.0005; table S2) switch to 67% of glaciers advancing (median rate, 20.0 m year^−1^). This trend continued into the most recent epoch (2000–2012), when 65% of glaciers advanced at a median rate of 17.9 m year^−1^.

Regionally, trends in glacier retreat in the 1970s and 1980s (Fig. 1A) are most pronounced between Dronning Maud Land and Enderby Land [drainage basin 5 (DB5) to DB7] and between Oates Land and Wilkes Land (DB13 to DB15), where 74 and 79% of glaciers retreated, respectively (median rates, −34.4 and −21.9 m year^−1^). Between Queen Mary Land and Kemp Land (DB8 to DB12), there was a less obvious change, with 53% of glaciers retreating (median rate, 1.4 m year^−1^). During the periods 1990–2000 and 2000–2012, however, outlet glaciers within every drainage basin showed a dominant signal of advance (Fig. 1, B and C). The one exception is Wilkes Land (DB13), where 74% of glaciers retreated at a median rate of −63.6 m year^−1^ between 2000 and 2012 (Fig. 1C and Table 1). This represents a highly significant (*P* < 0.0005; table S2) switch from the 1990s, when 75% of glaciers in Wilkes Land advanced. This is the first demonstration that glacier terminus changes in the region are linked to recently observed trends in mass loss (*4*–*6*); that is, Wilkes Land is the only area of significant mass loss in East Antarctica and the only area where outlet glaciers are retreating. We now turn our attention to analyzing the potential causes of this anomalous retreat, focusing on atmospheric warming, changes in ocean conditions, and alterations in sea-ice patterns.

**Table 1:** Glacier terminus position change across the entire EAIS at each epoch. Results from DB16 to DB13 were obtained from a previous study (*11*).

**Sample**	**1974–1990**		**1990–2000**		**2000–2012**	
	***n***	**Median (m year^−1^)**	***n***	**Median (m year^−1^)**	***n***	**Median (m year^−1^)**
**All glaciers**	254	−17.8	334	20.0	342	17.9
**DB16**	18	7.5	19	1.8	16	10.9
**DB15**	69	−3.5	70	12.6	71	20.6
**DB14**	24	−43.5	35	30.3	37	19.4
**DB13**	15	−49.4	37	54.9	39	−63.6
**DB12**	33	−3.4	40	8.9	41	15.9
**DB8/DB9/DB11**	19	−1.4	34	14.2	35	13.8
**DB7**	43	−38.0	60	24.5	62	29.1
**DB6/DB5**	15	−66.1	18	158.7	20	72.4
**DB4**	18	−23.6	21	54.5	21	49.9

**Fig. 2:**
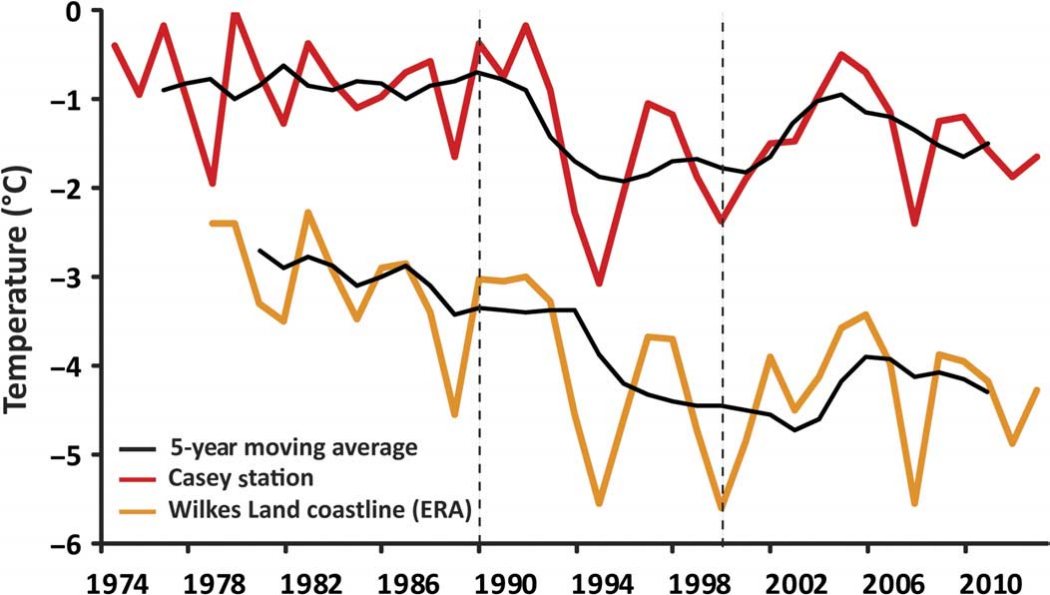
Mean austral summer (December to February) air temperature from Casey station (red) and the entire Wilkes Land coastline (orange) (sourced from the ERA-Interim data set).

In the Antarctic Peninsula, atmospheric warming has been linked to the ongoing retreat of glaciers (*13*) and the disintegration of ice shelves through excess surface meltwater driving hydrofracturing (*14*) and increased basal melt (*15*). Mean austral summer (December to February) temperature records in Wilkes Land show a period of relatively warm temperatures between 1974 and 1990, a cooling in the 1990s, and a slight increase in mean temperature between 2000 and 2012, although not to the same levels as in the 1970s and 1980s (Fig. 2). These temperature patterns are broadly consistent with the observed trends in glacier terminus position in Wilkes Land, raising the possibility that the observed changes might be driven by air temperatures. Moreover, although the mean monthly air temperatures are relatively cold (<0°C), surface temperature can still climb above freezing on a daily basis and will likely produce some surface melt (*16*). However, the amount of surface melt produced in Wilkes Land is considerably lower than that produced in other regions of Antarctica (*16*) (for example, the Antarctic Peninsula), where surface melt–induced hydrofracture has been a proposed mechanism of glacier retreat (*14*). Thus, we suggest that surface melt is unlikely to have driven the anomalous retreat of outlet glaciers in Wilkes Land. Rather, we suggest that changes in the ocean system (sea-ice and ocean temperatures) might play a predominant role, but these are nevertheless likely to be linked to changes in air temperature trends.

Ocean-driven basal melt can enhance iceberg calving (*17*) and ultimately influence glacier terminus positions. For example, in the Amundsen Sea, West Antarctica, the retreat (*18*) and increased discharge from outlet glaciers have been linked to increased upwelling of warm Circumpolar Deep Water (CDW) across the continental shelf break (*19*, *20*). Rates of basal melting in Wilkes Land are comparable to those of some West Antarctic ice shelves (*21*, *22*), but limited observational records indicate that it is unclear whether this is attributable to CDW, despite having been hypothesized (*22*).

**Fig. 3:**
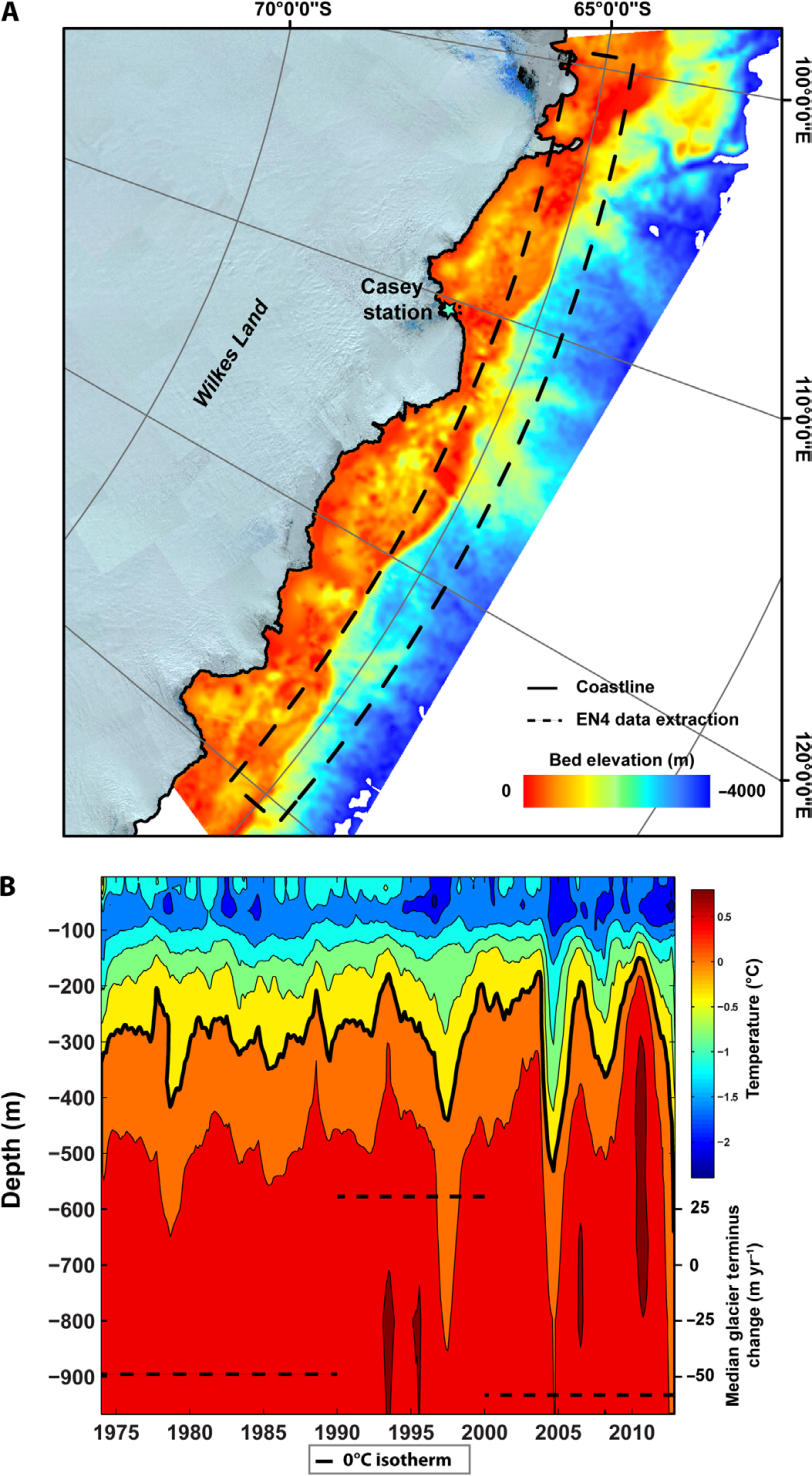
EN4 subsurface ocean temperature. (**A**) Bed topography of Wilkes Land (from Bedmap2), with the spatial location of the EN4 data extraction on the continental shelf boundary. (**B**) EN4 subsurface objective analysis temperature depth profile for 1974–2012. Data were extracted from 31 grid cells between 100° and 130°E, 64°S (continental shelf boundary, Wilkes Land), with the black dashed line representing the median glacier terminus position change of outlet glaciers per epoch in Wilkes Land.

In the absence of long-term observational records of ocean temperatures for Wilkes Land, we used EN4 subsurface ocean temperature objective analysis data (*23*) to gain plausible insight into potential changes in the upwelling of CDW at the continental shelf boundary since 1974 (Fig. 3, A and B). We acknowledge that the nature of these data creates very high uncertainty estimates (fig. S2), meaning that trends cannot be considered statistically significant. However, these data provide the only indication of possible changes in ocean temperatures in the absence of direct observations, and we include them to simply examine any possible trends that might be consistent with the glacier changes we observe. Estimates from EN4 indicate a cooling trend in the top 109 m of the water column (0.084°C per decade; fig. S2A), a warming trend at depths between 109 and 446 m (0.036°C per decade; fig. S2B), and no trend at depths between 446 and 967 m (fig. S2C). Despite the large uncertainties with the EN4 data, we also note that the only observational records that exist in this region (*24*) suggest the presence of modified CDW (mCDW) at depths similar to the warming trend between 109 and 446 m on the continental shelf break in Wilkes Land. Moreover, the potential discovery of an inland trough connecting Totten glacier cavity to the ocean in this region (*25*) increases the likelihood of mCDW crossing the continental shelf boundary and enhancing basal melt. However, although the EN4 data might hint at a long-term warming trend at intermediate depths, this is not entirely consistent with the longer-term trend in glacier terminus position; that is, glacier advance in the 1990s deviates from the potential warming trend. Thus, we suggest that processes more local to the glacier termini may modulate whether the warm waters can access the glacier terminus and sea-ice processes are known to modify ocean stratification.

**Fig. 4:**
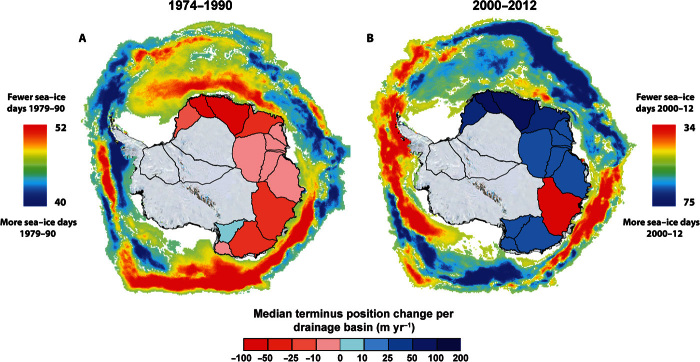
Difference in the number of sea-ice days per year during the sea-ice season (April to October) compared to 1990–2000, with shaded drainage basins representing glacier terminus position trends (red, retreat; blue, advance). (**A** and **B**) Data for (A) 1974–1990 and (B) 2000–2012. Sea-ice data only extend back to 1979 (see Materials and Methods).

Differences in the number of sea-ice days per year (April to October) between each epoch reveal clear spatial trends across Antarctica (Fig. 4). In East Antarctica, there were 2.5 fewer sea days per year on average in the period 1979–1990 compared to the 1990s (fig. S3). Negative anomalies were concentrated between Oates Land (DB15) and Queen Mary Land (DB12), and between Enderby Land (DB7) and Coats Land (DB4). In both regions, there was a dominant signal of glacier retreat between 1974 and 1990 (Fig. 4A), with a mean reduction in sea ice of 11.9 and 7.0 days per year, respectively. However, between Princess Elizabeth Land (DB12) and Kemp Land (DB8), there were 1.4 more sea-ice days per year on average in the period 1979–1990 than in the 1990s. Here, there was little change in glacier terminus position between 1974 and 1990 (Fig. 4A and Table 1).

More recently, there has been only a small change in the overall average number of sea-ice days per year between 2000 and 2012, compared to the 1990s (Fig. 4B). However, there is considerable spatial variability, with an average increase of 5.3 days per year (maximum of 31 days per year) in the outer sea-ice pack off the coast of Oates Land, eastern Dronning Maud Land, and Kemp Land, where there was also a signal of glacier advance between 2000 and 2012 (Fig. 4B). However, in Wilkes Land, where 74% of glaciers retreated between 2000 and 2012, there was an average decrease of 11.5 sea-ice days per year (maximum of −27 days per year) (Fig. 4B). Thus, patterns in sea-ice change correspond to changing patterns in glacier terminus position.

**Fig. 5:**
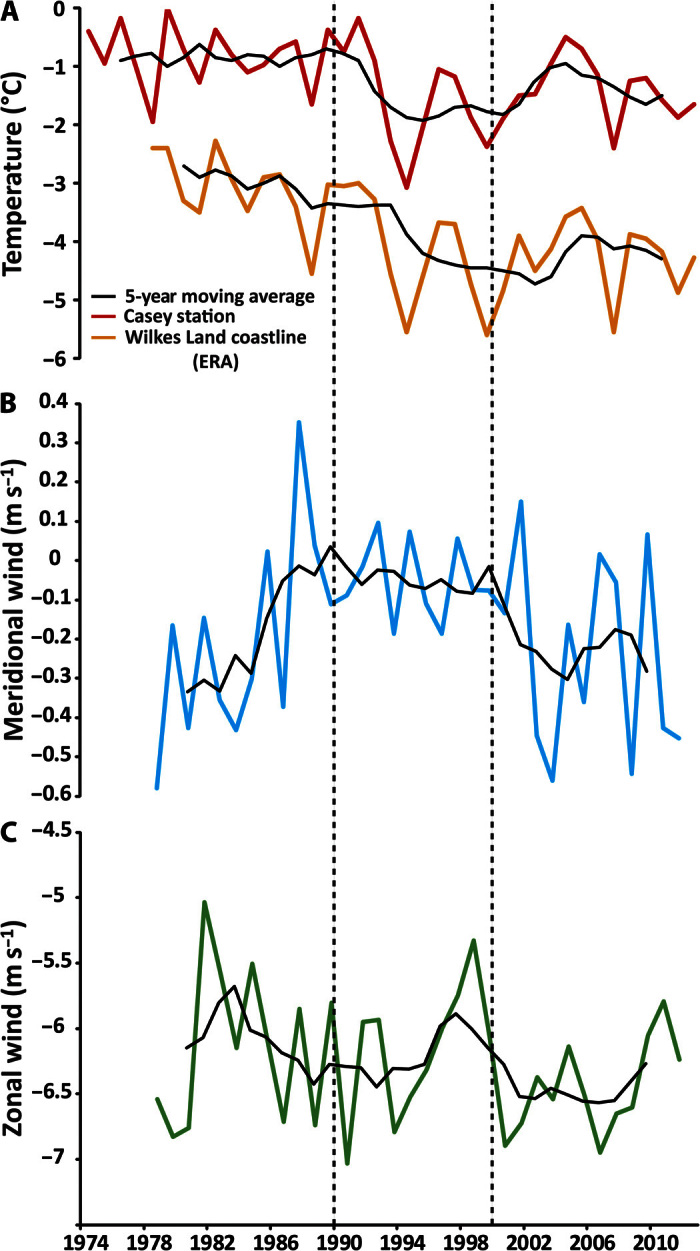
Time series of winter air temperature and meridional wind flow. (**A**) Mean winter (April to October) air temperature from Casey station (red) and the entire Wilkes Land coast from the ERA-Interim data set (orange). (**B**) Mean monthly meridional flow on the Wilkes Land coast; positive values indicate southerly winds (south to north). (**C**) Mean monthly zonal flow on the Wilkes Land coast; positive values indicate westerly winds (west to east).

Coastal polynyas are important in driving sea-ice variability in Antarctica. They produce around 10% of total sea ice, despite representing only 1% of the maximum sea-ice area (*26*). These polynyas form through katabatic winds and are thought to be sensitive to small changes in climate (*27*). In Wilkes Land, there are major negative sea-ice anomalies in 1979–1990 and 2000–2012, relative to 1990–2000. Analysis of mean ERA-Interim (*28*) meridional flow, zonal flow, and air temperatures on the coastline of Wilkes Land during the sea-ice production season (April to October) suggests a tendency for more northerly winds and higher temperatures during periods of negative sea-ice anomalies (1979–1990 and 2000–2012), with no clear change in zonal flow (Fig. 5). We suggest that this likely represents a suppression in katabatic winds that, in turn, decreases coastal polynya intensity and results in a decrease in sea-ice production in Wilkes Land in 1974–1990 and 2000–2012, relative to the 1990s.

Changes in the amount of sea-ice production can alter the mixing of shelf waters, which can drive variability in the basal melt rate of glacier termini (*29*–*31*). Wilkes Land is a region of intense wind-driven coastal polynya activity, which (as a consequence) has high rates of sea-ice production (*26*). During sea-ice production, brine rejection leads to the deepening of the cold surface mixed layer and to the increased destratification of the water column (*32*). The depth of the mixed layer is intrinsically linked to the net salt flux, which is controlled by the amount of sea ice produced, with higher rates of sea-ice production on the continental shelf resulting in a deeper mixed layer (*32*). In some medium to large polynyas in Wilkes Land, models have suggested that sufficient sea-ice production can cause the complete destratification of the water column (that is, the mixed layer extends to the sea bed), resulting in the formation of High-Salinity Shelf Water (HSSW) (*33*); direct observations have confirmed this in the Vincennes Bay polynya (*34*). HSSW is important because it is the densest and coolest water mass on the continental shelf and its presence can prevent the less dense and warmer mCDW from directly accessing glacier cavities, thus suppressing basal melt. Therefore, any variation in sea-ice production and the subsequent supply of HSSW have the potential to influence the basal melt rate of glaciers (fig. S4). This is confirmed by recent reports that directly link interannual variations in the sea-ice production of the Dalton polynya to the basal melt rate of the nearby Totten glacier in Wilkes Land (*29*, *31*).

We therefore hypothesize that the recent reduction in sea-ice days in Wilkes Land (2000–2012; Fig. 4) has led to decreased brine rejection and thus a decreased supply of HSSW. This, combined with a possible longer-term underlying trend of increased entrainment of CDW at the continental shelf boundary (Fig. 3), has increased the likelihood of warm mCDW reaching glacier cavities, enhancing basal melt and driving glacier retreat. This is consistent with the reported high rates of basal melt (*21*, *22*) and mass loss (*4*–*6*) in Wilkes Land during the same period. The relationship we identified between glacier trends and changes in the number of sea-ice days in each year is consistent throughout the study period in Wilkes Land, with positive sea-ice anomalies from 1990 to 2000 corresponding to glacier advance during the same period and negative sea-ice anomalies from 1979 to 1990 corresponding to glacier retreat. Indeed, the close relationship between glacier terminus position and sea-ice conditions around the entire EAIS (Fig. 4) raises the possibility that this process may be driving glacier terminus position trends in other areas of high sea-ice production in the EAIS.

We note that the only regions of Antarctica with similar large reductions in sea-ice days per year to those observed offshore of Wilkes Land are the Amundsen Sea sector of West Antarctica and the west coast of the Antarctic Peninsula (Fig. 4). The Amundsen Sea sector has long been recognized as West Antarctica’s “weak underbelly” (*7*), where glacier retreat (*18*) and mass loss (*4*–*6*) have been linked to CDW driving marine ice-sheet instability. Our results suggest that a similar scenario is emerging in Wilkes Land, which is the only region of East Antarctica where outlet glaciers are both losing mass (*4*–*6*) and retreating during the past decade, as our data show. However, whereas the retreat and mass loss of glaciers in the Amundsen Sea sector has been linked to wind-driven upwelling of CDW across the continental shelf (*19*, *20*), we hypothesize that the retreat of glaciers in Wilkes Land is linked to a reduction in sea-ice production, which is consistent with meridional wind patterns. Given the potential sea level contribution of outlet glaciers in Wilkes Land, there is an urgent need for further integration of glaciological and oceanographic data to inform numerical modeling and constrain future predictions of this potentially weak underbelly of East Antarctica.

## MATERIALS AND METHODS

### Glacier terminus mapping

We used Landsat imagery from the Multispectral Scanner (MSS), Thematic Mapper (TM), and Enhanced Thematic Mapper Plus (ETM+) satellites to map the terminus position of 176 outlet glaciers along the coast of East Antarctica across four approximate time steps: 1974, 1990, 2000, and 2012, stretching from Queen Mary Land to the Ronne Ice Shelf. This extends the record of a previous study (*11*) to create a data set covering the entire EAIS. The change in glacier terminus position was calculated as the area change at each time step divided by the width, which was obtained by a reference box that approximately delineated the sides of the glacier (*35*). The accuracy of the mapping was limited by the coregistration of Landsat mosaics to the 2000 base image. This resulted in the following estimates of inaccuracies in mapping: ±60 m (2010), ±180 m (1990), and ±250 m (1974). These are sufficient for extracting the decadal trends we present in this study, and we note that most changes lie well outside these uncertainties.

### EN4 subsurface ocean reanalysis

We use the EN4.0.2 subsurface ocean temperature objective analysis data set (*23*), which is available at the UK Meteorological Office Hadley Centre (www.metoffice.gov.uk/hadobs/en4/download-en4-0-2.html). The objective analysis data set covers the entire study period on a monthly basis and is available at a 1° × 1° spatial resolution, with data obtained from the WOD09, GTSPP, Argo, and ASBO collections (*23*). The mean temperature value of the 31 grid cells covering the continental shelf boundary in Wilkes Land (Fig. 3A) was calculated for each month during the study period (1974–2012) and at 26 different depths (5, 15, 25, 35, 45, 55, 66, 76, 87, 98, 109, 121, 135, 149, 165, 184, 207, 235, 270, 315, 372, 446, 540, 657, 799, and 967 m). Uncertainty estimates for the EN4 data set are included in fig. S2 and are also available at the UK Meteorological Office Hadley Centre (www.metoffice.gov.uk/hadobs/en4/download-en4-0-2.html).

### Sea ice

We used the Bootstrap sea-ice concentrations derived from the Nimbus-7 satellite and Defense Meteorological Satellite Program (DMSP) satellites (*36*) to calculate trends in the number of sea-ice days per year around Antarctica (http://nsidc.org/data/nsidc-0079). The data set offers near-complete coverage of the Antarctic sea-ice zone on a daily basis since July 1987 and every 2 days before this stretching back to October 1978 at a spatial resolution of 25 km × 25 km. There are brief gaps in the data set in August 1982 (4, 8, and 16 August) and 1984 (13 to 23 August). These have been interpolated where missing pixels were present (see http://nsidc.org/data/nsidc-0079).

In line with other studies (*37*), a sea-ice day was classified as a grid cell with a sea-ice concentration greater than 15%. The total number of sea-ice days was calculated for each year during the sea-ice season (April to October), with sea-ice days doubled between April 1979 and July 1987 to account for the 2-day temporal resolution of the satellites during that period. The mean number of sea-ice days per year for each epoch was calculated before calculating the difference in sea-ice days between epochs (for example, Fig. 4).

### Climate data

Monthly mean surface air temperature records from Casey station were extracted from the Scientific Committee on Antarctic Research (SCAR) Met-READER project (https://legacy.bas.ac.uk/met/READER/). In addition, mean monthly 0.25° ERA-Interim (*28*) 2-m air temperature and 10-m meridional and zonal wind (http://apps.ecmwf.int/datasets/data/interim-full-moda/levtype=sfc/) from 1979 to 2012 were extracted from the Wilkes Land coastline. For the purpose of this study, we defined the Wilkes Land coastline as a shapefile consisting of the coastline of DB13 with a 25-km buffer.

## Supplementary Material

http://advances.sciencemag.org/cgi/content/full/2/5/e1501350/DC1

## SUPPLEMENTARY MATERIALS

Supplementary material for this article is available at http://advances.sciencemag.org/cgi/content/full/2/5/e1501350/DC1 fig. S1. A series of mapping figures, with digitized terminus positions (green, 1974; yellow, 1990; blue, 2000; red, 2012) and glacier ID numbers, overlain on the 2000 Landsat base image. fig. S2. Subsurface ocean change per meter in DB13 with uncertainty estimates (for example, Fig. 3). fig. S3. Mean winter (April to October) sea-ice days for 1990–2000 (for example, the reference period in Fig. 4). fig. S4. Schematic diagram of shelf water dynamics in Wilkes Land. table S1. Glacier terminus position change across each epoch. table S2. Wilcoxon tests for significant differences between glacier terminus position change between each epoch. database S1. Terminus position change measurements for all outlet glaciers in East Antarctica.
