# Supplementary material for: Pan–ice-sheet glacier terminus change in East Antarctica reveals sensitivity of Wilkes Land to sea-ice changes
Source: Sci Adv. 2016 May 6;2(5):e1501350. doi: 10.1126/sciadv.1501350 (PMC4928901; doi:10.1126/sciadv.1501350)
Supplement: http://advances.sciencemag.org/cgi/content/full/2/5/e1501350/DC1 [file supp_2_5_e1501350__index.html]

Science Advances | Science Advances

## Supplementary Materials

**This PDF file includes:**

- fig. S1. A series of mapping figures, with digitized terminus positions (green, 1974; yellow, 1990; blue, 2000; red, 2012) and glacier ID numbers, overlain on the 2000 Landsat base image.
- fig. S2. Subsurface ocean change per meter in DB13 with uncertainty estimates (for example, Fig. 3).
- fig. S3. Mean winter (April to October) sea-ice days for 1990–2000 (for example, the reference period in Fig. 4).
- fig. S4. Schematic diagram of shelf water dynamics in Wilkes Land.
- table S1. Glacier terminus position change across each epoch.
- table S2. Wilcoxon tests for significant differences between glacier terminus position change between each epoch.
- database S1. Terminus position change measurements for all outlet glaciers in East Antarctica.

Download PDF

**Files in this Data Supplement:**

- Adobe PDF - 1501350\_SM.pdf
