# Supplementary material for: Pan–ice-sheet glacier terminus change in East Antarctica reveals sensitivity of Wilkes Land to sea-ice changes
Source: Sci Adv. 2016 May 6;2(5):e1501350. doi: 10.1126/sciadv.1501350 (PMC4928901; doi:10.1126/sciadv.1501350)
Supplement: http://advances.sciencemag.org/cgi/content/full/2/5/e1501350/DC1 [file 1501350_SM.pdf]

Science Advances

AAAS

advances.sciencemag.org/cgi/content/full/2/5/e1501350/DC1

Supplementary Materials for

Pan–ice-sheet glacier terminus change in East Antarctica reveals sensitivity of Wilkes Land to sea-ice changes

Bertie W. J. Miles, Chris R. Stokes, Stewart S. R. Jamieson

Published 6 May 2016, *Sci. Adv.* **2**, e1501350 (2016)
DOI: 10.1126/sciadv.1501350

The PDF file includes:

- fig. S1. A series of mapping figures, with digitized terminus positions (green, 1974; yellow, 1990; blue, 2000; red, 2012) and glacier ID numbers, overlain on the 2000 Landsat base image.
- fig. S2. Subsurface ocean change per meter in DB13 with uncertainty estimates (for example, Fig. 3).
- fig. S3. Mean winter (April to October) sea-ice days for 1990–2000 (for example, the reference period in Fig. 4).
- fig. S4. Schematic diagram of shelf water dynamics in Wilkes Land.
- table S1. Glacier terminus position change across each epoch.
- table S2. Wilcoxon tests for significant differences between glacier terminus position change between each epoch.
- database S1. Terminus position change measurements for all outlet glaciers in East Antarctica.

Supplementary Materials

Supplementary Figures

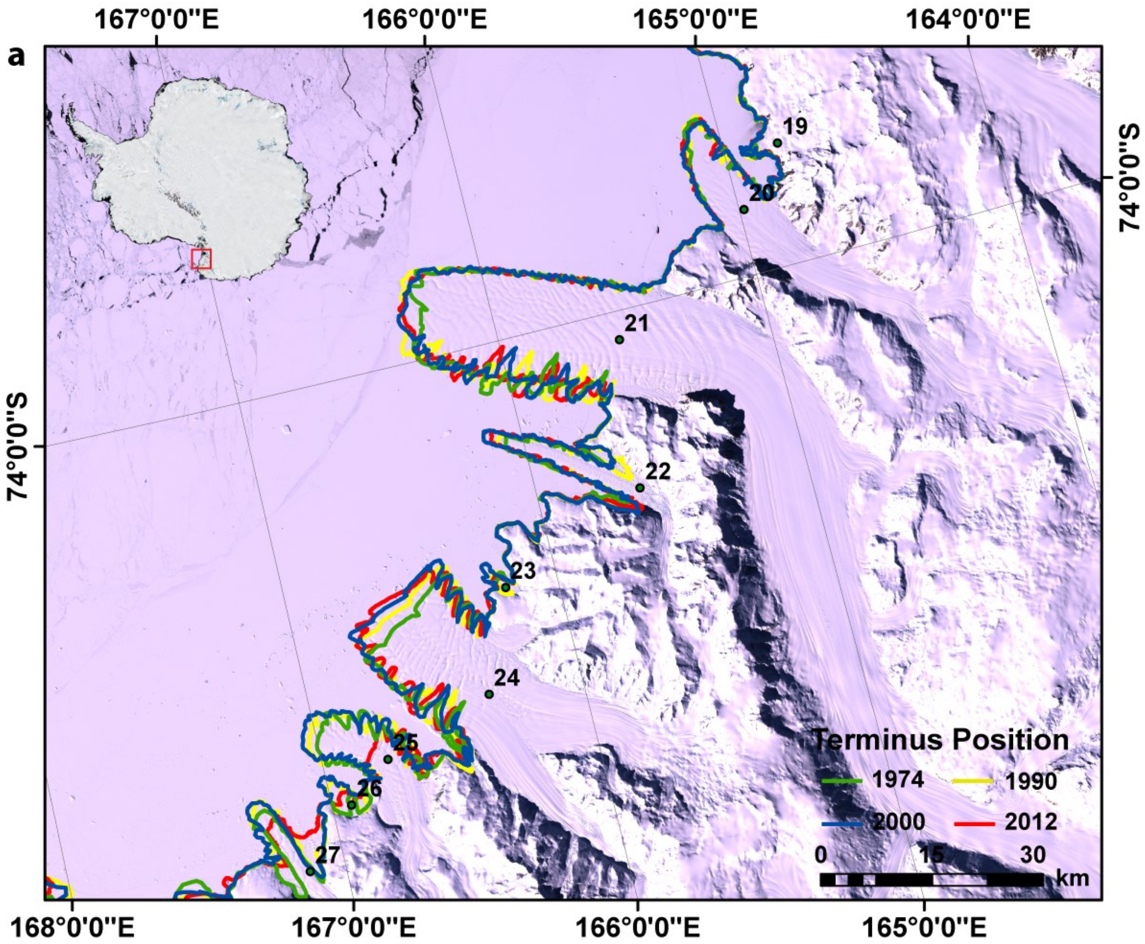

b

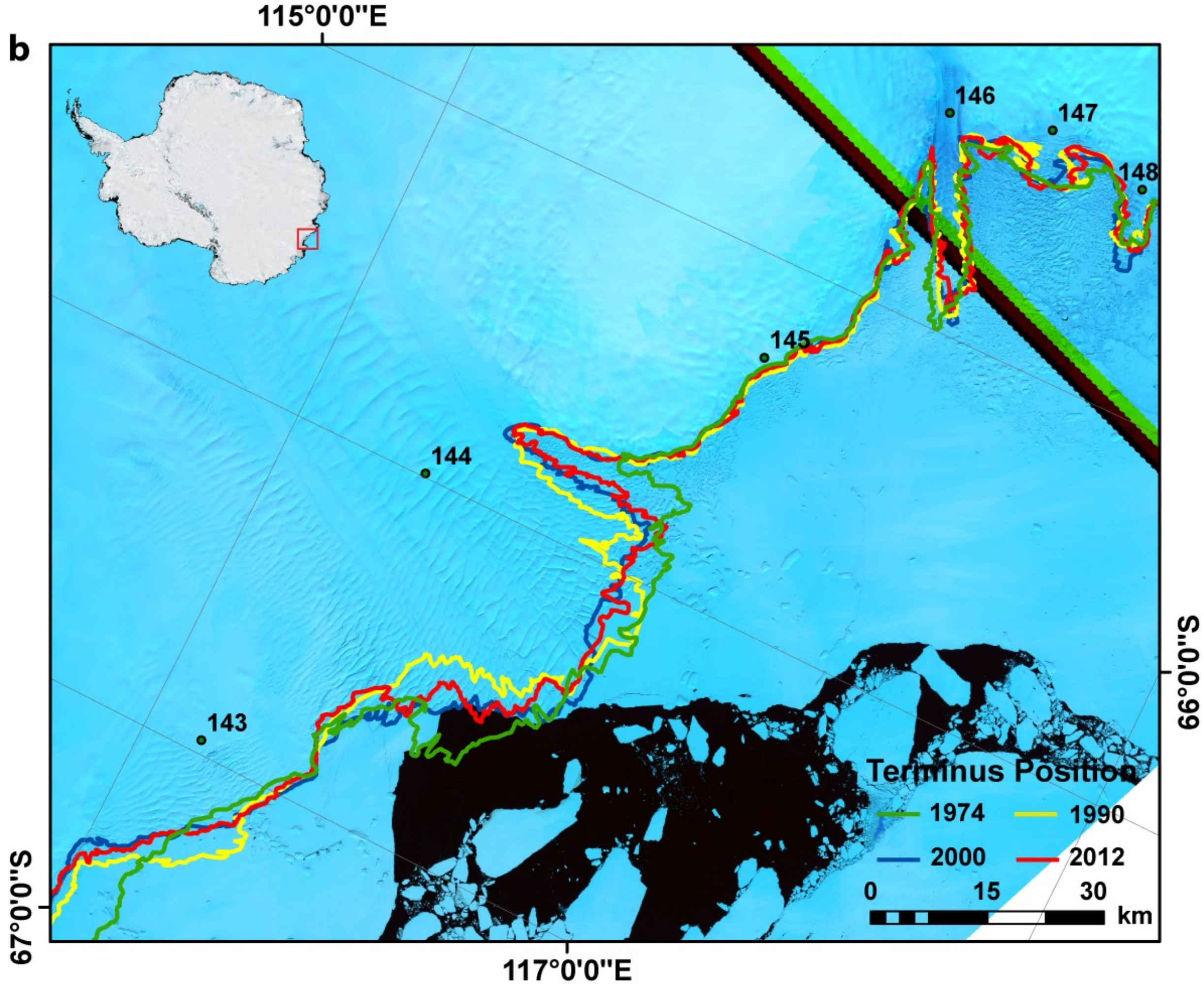
115°0'0"E
117°0'0"E
66°0'0"S
67°0'0"S
143
144
145
146
147
148
Terminus Position
1974
1990
2000
2012
0
15
30
km

c

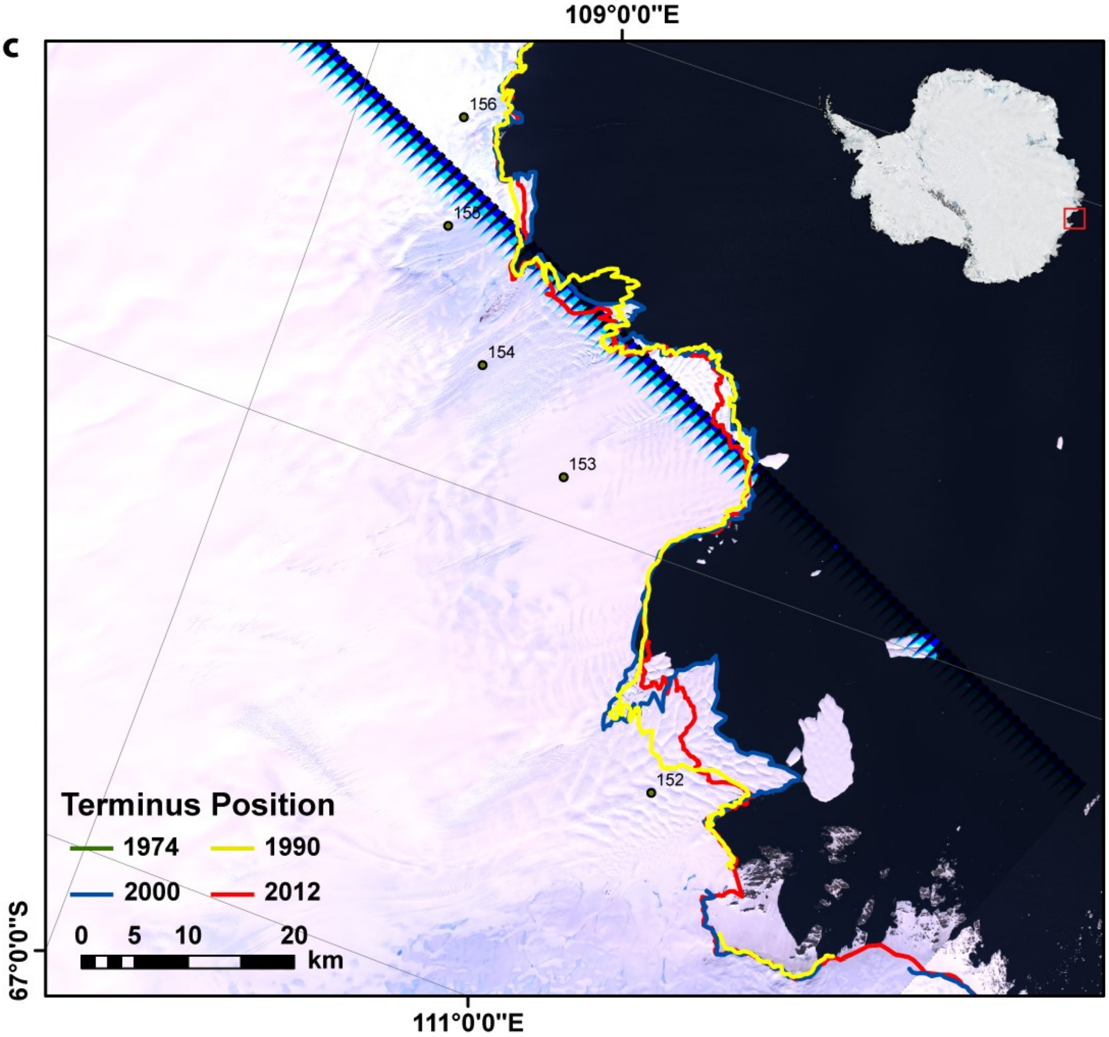
109°0'0"E
111°0'0"E
67°0'0"S
152
153
154
155
156
Terminus Position
1974
1990
2000
2012
0
5
10
20
km

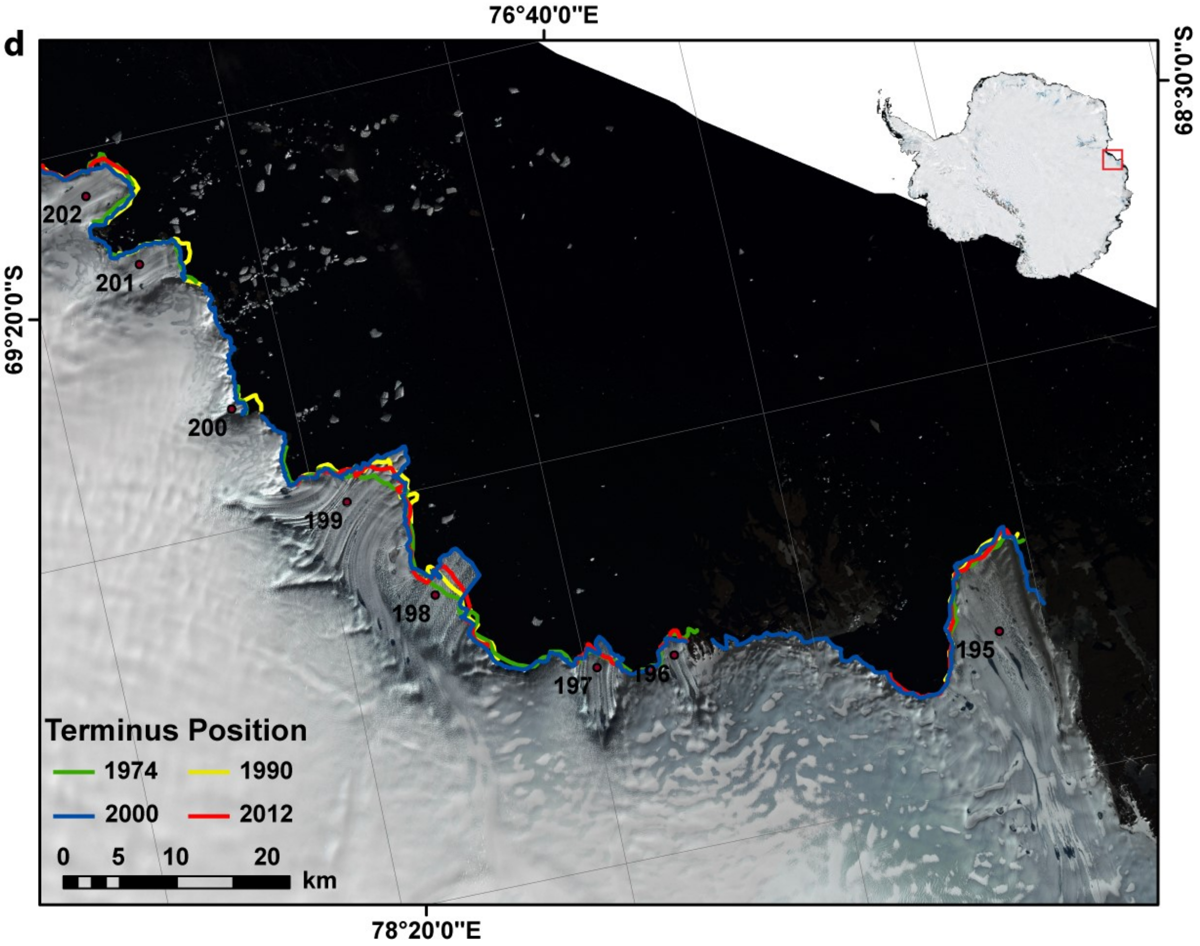
d
76°40'0"E
68°30'0"S
69°20'0"S
78°20'0"E
202
201
200
199
198
197
196
195
Terminus Position
1974
1990
2000
2012
0 5 10 20
km

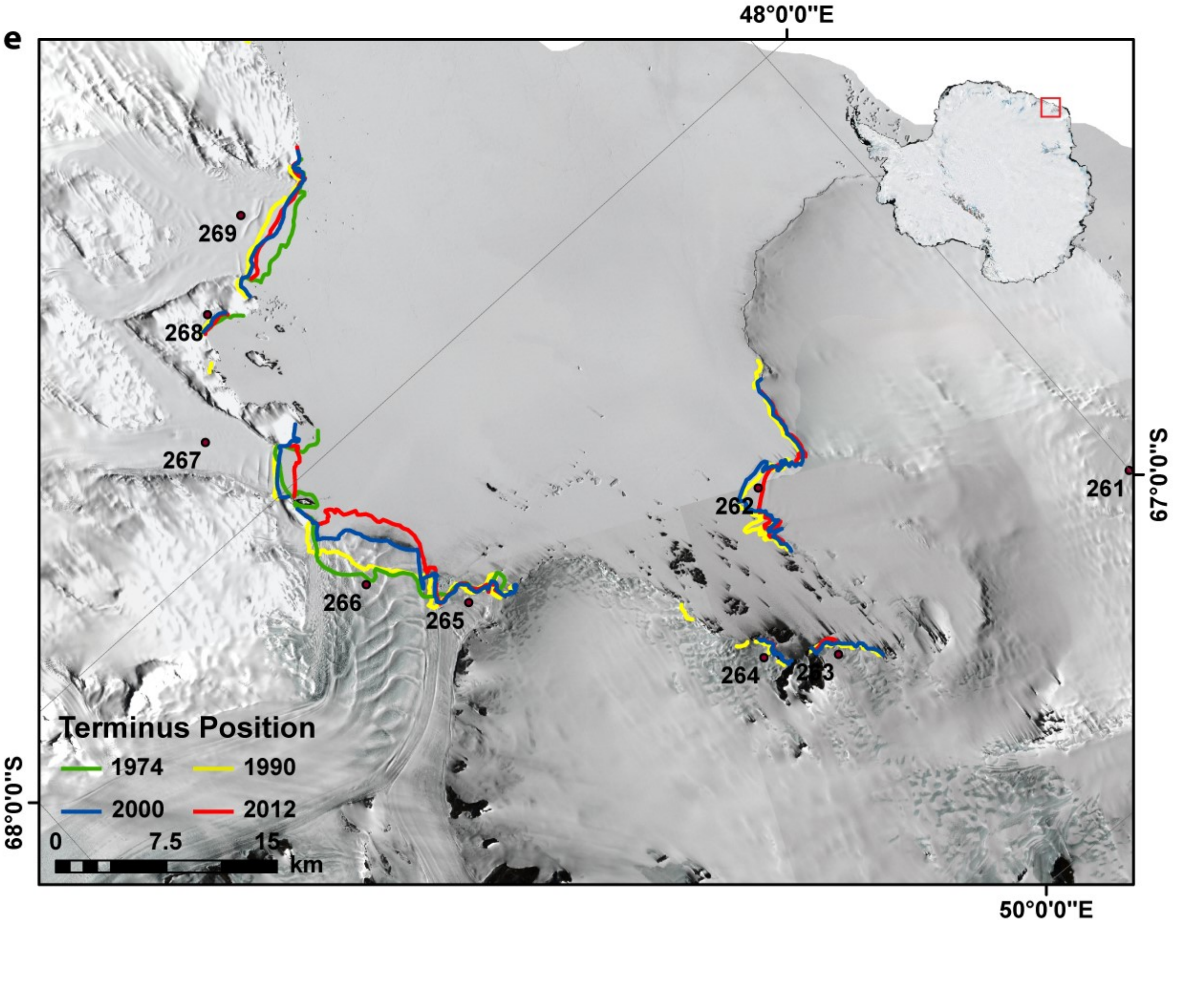
e
48°0'0"E
67°0'0"S
68°0'0"S
50°0'0"E
269
268
267
266
265
264
263
262
261
Terminus Position
1974
1990
2000
2012
0 7.5 15
km

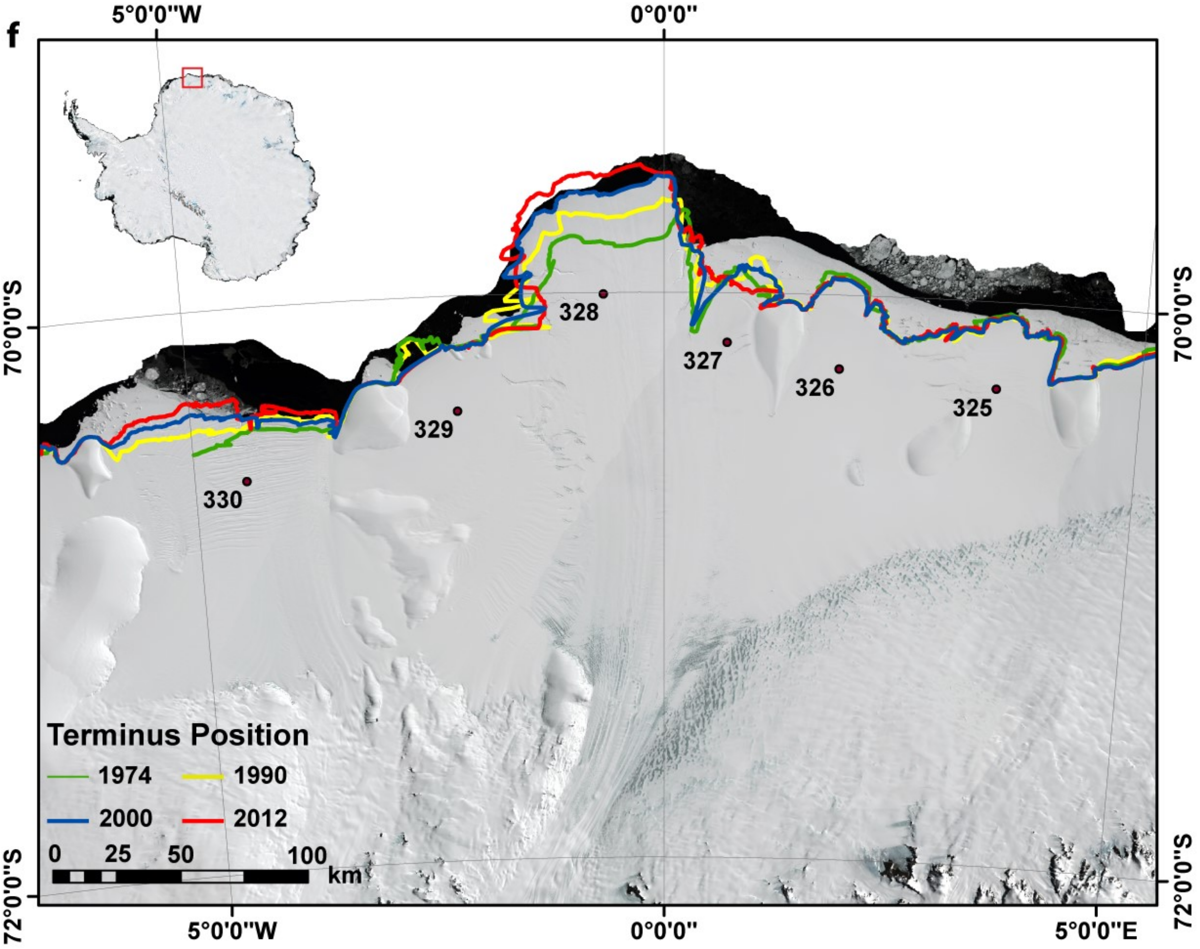

fig. S1. A series of mapping figures, with digitized terminus positions (green, 1974; yellow, 1990; blue, 2000; red, 2012) and glacier ID numbers, overlain on the 2000 Landsat base image. (**a**) Victoria Land (DB16) (**b** and **c**) Wilkes Land (DB13) (**d**) Queen Mary Land (DB12) (**e**) Enderby Land (DB7) (**f**) Dronning Maud Land (DB 5 and 6).

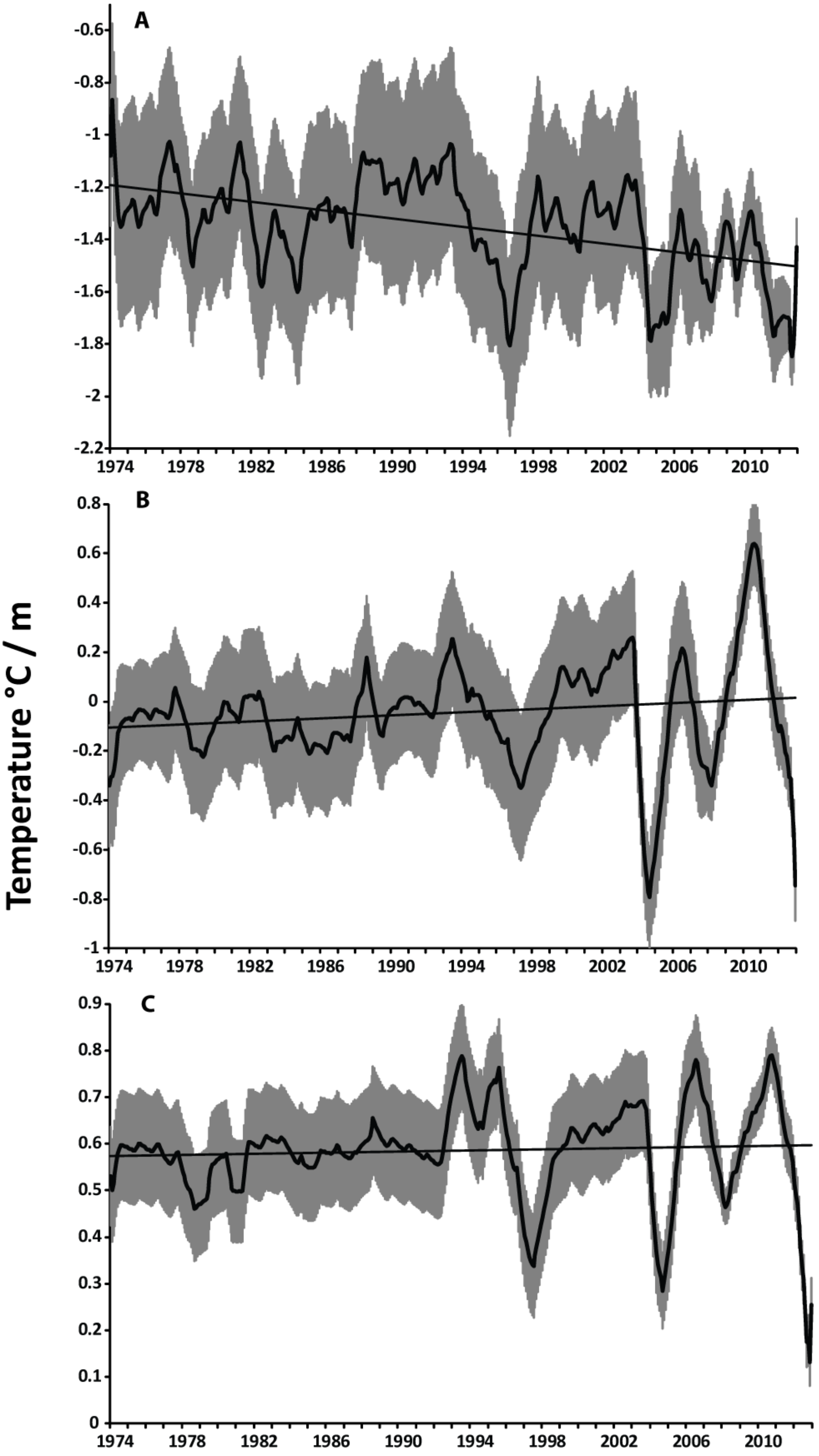

fig. S2. Subsurface ocean change per meter in DB13 with uncertainty estimates (for example, Fig. 3). (**a**) 5-109 m (**b**) 109-446 m (**c**) 446-967 m.

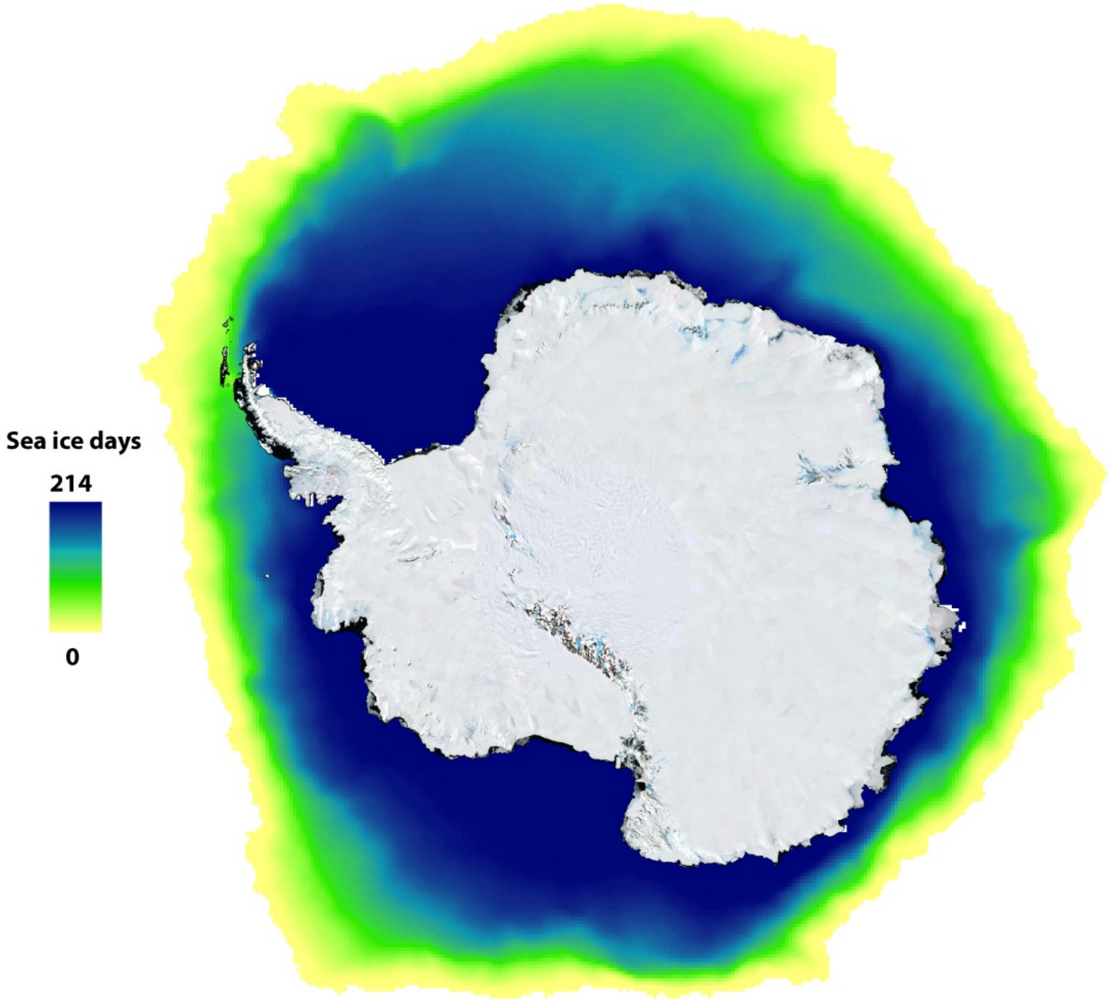

fig. S3. Mean winter (April to October) sea-ice days for 1990–2000 (for example, the reference period in Fig. 4).

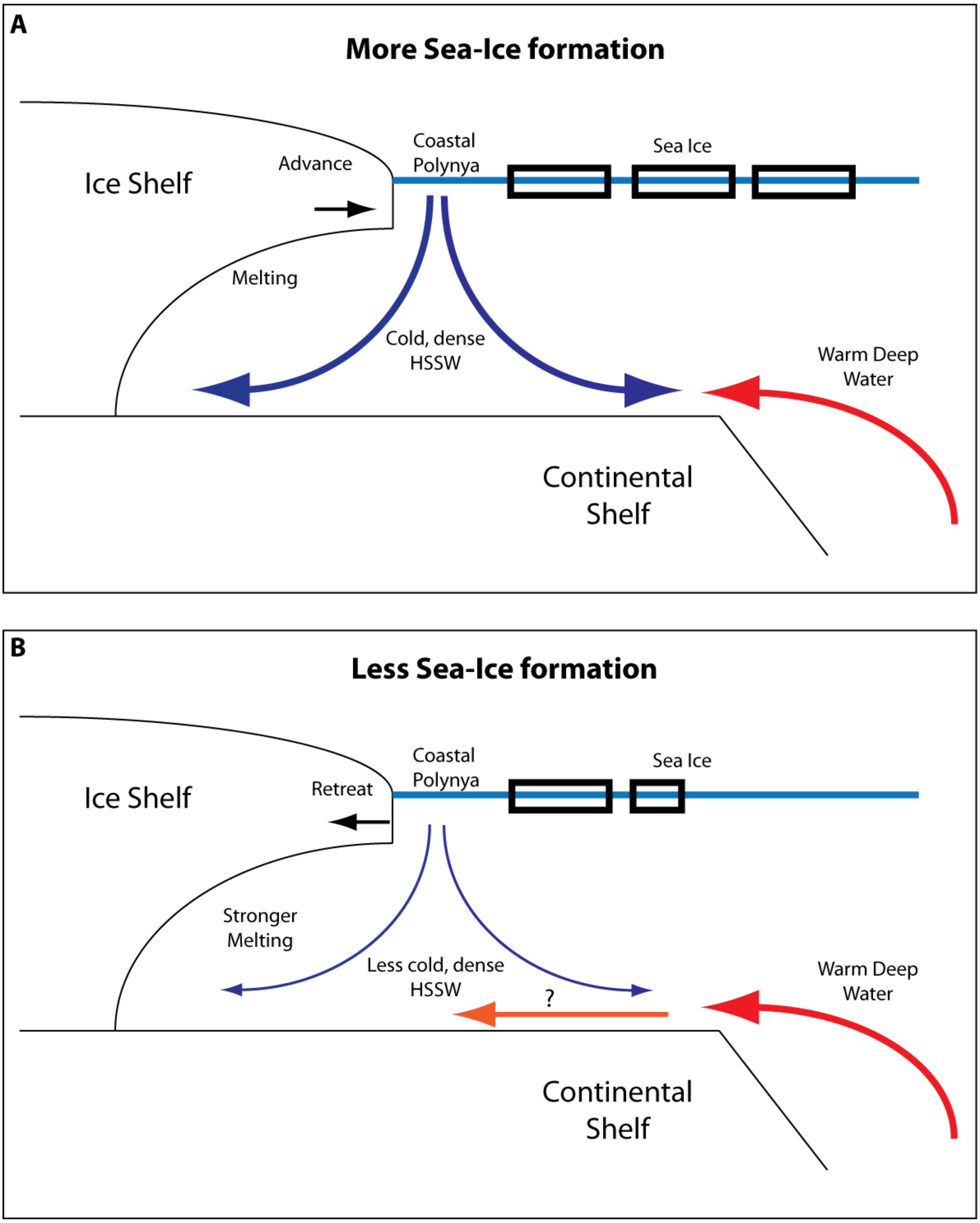

fig. S4. Schematic diagram of shelf water dynamics in Wilkes Land. (A) More sea-ice formation (1990s): increase sea ice production results in a greater supply of HSSW, resulting in less basal melt and glacier advance. (**B**) Less sea-ice formation (1974-1990; 2000-2012): Less sea-ice formation results in less HSSW, stronger basal melt and glacier retreat.

Supplementary Tables

table S1. Glacier terminus position change across each epoch.

Epoch	n =	Advance (%)	Retreat (%)	median (m yr^{-1})
**1974-2012**	262	50	50	-0.4
**1974-1990**	254	35	65	-17.8
**1990-2000**	334	67	33	20.0
**2000-2012**	342	65	35	17.9

table S2. Wilcoxon tests for significant differences between glacier terminus position change between each epoch.

Sample	Epoch	N=	Median	P=
**All Glaciers**	1974-1990	254	-17.8	**0.0000**
	1990-2000	334	20.0	
	1990-2000	334	20.0	0.1314
	2000-2012	342	17.9	
**DB16**	1974-1990	18	7.5	0.7382
	1990-2000	19	1.8	
	1990-2000	19	1.8	0.8167
	2000-2012	16	10.9	
**DB15**	1974-1990	69	-3.5	**0.0001**
	1990-2000	70	12.6	
	1990-2000	70	12.6	0.8787
	2000-2012	71	20.6	
**DB14**	1974-1990	24	-43.5	**0.0001**
	1990-2000	35	30.3	
	1990-2000	35	30.3	0.7999
	2000-2012	38	19.4	
**DB13**	1974-1990	15	-49.4	**0.0002**
	1990-2000	37	54.9	
	1990-2000	37	54.9	**0.0000**
	2000-2012	39	-63.6	
**DB12**	1974-1990	33	-3.4	0.2014
	1990-2000	40	8.9	

	1990-2000	40	8.9	
	2000-2012	41	15.9	0.8386
DB8/DB9/DB11	1974-1990	19	-1.4	
	1990-2000	34	14.2	**0.0438**
	1990-2000	34	14.2	
	2000-2012	35	13.8	0.4545
DB7	1974-1990	43	-38.0	
	1990-2000	60	24.5	**0.0000**
	1990-2000	60	24.5	
	2000-2012	62	29.1	0.7648
DB6/DB5	1974-1990	15	-66.1	
	1990-2000	18	158.7	0.1118
	1990-2000	18	158.7	
	2000-2012	20	72.4	0.0854
DB4	1974-1990	18	-23.6	
	1990-2000	21	54.5	**0.0139**
	1990-2000	21	54.5	
	2000-2012	21	49.9	0.3392

database S1. Terminus position change measurements for all outlet glaciers in East Antarctica. Note: terminus position change data from ID 1-175 were obtained from a previous study[11]

[1]ID	[2]Name	[3]Width (km)	Lat	Lon	[4]Drainage Basin	[5]Terminus position change (m a^{-1})		
						1974-1990	1990-2000	2000-2010
1	Ferrar	6.04	-77.681	163.273	16	-48.86	-32.82	30.44
2	Debenham	8.2	-77.109	163.158	16	-26.47	-43.76	
3	-	0.94	-76.993	162.904	16	6.05	8.46	
4	New	1.14	-76.994	162.401	16			
5	Mackay	3.46	-76.936	162.225	16	93.77	-32.71	
6	Hunt	0.87	-76.828	162.538	16	-47.1	1.77	8.69
7	Bens on	3.27	-76.798	162.563	16	-31.1	-2.99	15.12
8	Hedblom	5.65	-76.522	162.592	16		-0.72	18.06
9	Mawson	8.28	-76.142	162.420	16	184.64	187.26	207.04
10	Marin	2.7	-76.023	162.362	16	-11.65	-63.67	13.14
11	Harbord	3.63	-75.876	162.628	16	43.23	7.2	-26.02
12	-	6.23	-75.690	162.808	16	33.66	18.77	-28.49
13	Geikie	12.23	-75.493	162.719	16	27.49	-380.91	48.45
14	David	25.13	-75.347	163.322	16	517.95	528.8	-651.02
15	Larsen	5.1	-75.080	162.502	16	-87.65	119.55	-165.33
16	Reeves	7.84	-74.930	162.789	16	196.92	193.65	-778.23
17	Priestley	17.38	-74.949	163.322	16	114.12	132.8	747.71
18	Campbell	6.96	-74.510	164.378	16	-25.13	-35.45	-64.09
19	-	1.94	-74.078	164.794	16	8.98	9.9	-76.67
20	Tinker	5.77	-74.021	164.981	16	0.76	-0.98	22.87
21	Aviator	1.74	-73.923	165.559	15	341.38	-289.54	-216.41
22	Parker	2.84	-73.768	165.621	15	-45.81	129.33	45.84
23	-	1	-73.702	166.194	15	-39.44	193.01	-193.09
24	Icebreaker	12.86	-73.599	166.346	15	71.95	72.4	-22.27
25	Wylde	5.51	-73.557	166.762	15	69.78	46.61	-795.36
26	-	1.46	-73.520	166.929	15	231.34	173.09	-441.41
27	Suter	2.65	-73.462	167.131	15	223.82	-10.76	-86.07
28	Ridgeway	9.07	-73.368	167.339	15	78.5	38.01	-98.58
29	Mariner	16.88	-73.350	168.370	15	70.84	-10.53	40.07
30	Langevad	15.74	-73.269	168.748	15	-81.77	116.58	160.14
31	-	2.69	-73.105	169.203	15	-24.03	28.47	35.22
32	-	3.49	-73.070	169.265	15	-3.53	25.55	23.77
33	-	1.31	-73.063	169.371	15	-11.93	13.36	46.53
34	Tucker	8.54	-72.592	169.890	15	41.29	-50.99	155.74
35	Arneb	1.89	-72.390	170.028	15	36.87	-135.17	74.45
36	Edisto	2.8	-72.387	169.881	15	12.78	-44.2	2.45
37	Manhaul	3.59	-72.342	169.792	15	20.35	-25.18	44.12
38	-	1.79	-72.278	169.897	15	-0.18	3.28	-4.11
39	-	1.49	-72.223	169.893	15	1.83	-0.87	8.39
40	-	1.06	-72.213	169.866	15	1.14	0.75	7.41
41	-	1.4	-72.204	169.831	15	4.4	6.31	9.18
42	-	1.26	-72.188	169.757	15	4.66	-4.01	40.02
43	-	2.18	-72.152	169.719	15	79.8	27.65	20.74
44	Ironside	2.94	-72.120	169.828	15	-302.63	265.15	346.73
45	Burnette	2.62	-71.968	170.075	15	30.04	13.48	87.86
46	Moubray	9.5	-71.923	170.362	15	-17.8	100	115.45
47	Newnes	1.97	-71.622	170.235	15	-17.92	18.99	-3.81
48	Murray +	4.23	-71.550	169.936	15	-18.44	44	79.43
49	Egeberg	1.18	-71.504	169.833	15	-1.03	6.01	17.51
50	Nielsen	1.83	-71.462	169.679	15	-5.36	11.37	0.69
51	Ommanney	2.28	-71.471	169.484	15	-27.64	-19.75	143.14
52	Haffner	1.76	-71.410	169.422	15	2.05	7.52	38.65
53	Frank Newnes	0.65	-71.401	169.313	15	30.16	14.72	83.96
54	Shipley	1.4	-71.372	169.282	15	-13.05	5.06	24.79
55	-	3.74	-71.328	169.204	15	1.03	6.48	-3.41
56	-	0.82	-71.296	169.110	15	9.48	3.2	14.65

57	-	2.1	-71.289	169.008	15	-9.29	2.34	10.75
58	Frendley	2.45	-71.212	168.806	15	-23.36	11.86	133.35
59	Simpson	2.53	-71.195	168.735	15	7.69	0.37	10.02
60	-	2.32	-71.156	168.570	15	0.74	-1.73	6.17
61	Denis Toun	5.63	-71.098	168.161	15	21.06	4.69	124.62
62	-	2.67	-71.012	167.954	15	-0.58	8.62	2.17
63	Barnett	4.65	-70.918	167.748	15	-22.37	57	96.41
64	-	0.84	-70.789	167.780	15	5.43	-2.04	14.18
65	-	0.92	-70.734	167.415	15	-37.76	20.59	27.37
66	-	0.77	-70.715	167.289	15	-0.29	6.01	-11.54
67	Fortenberry	1.21	-70.734	166.958	15	0.49	-7.92	6.89
68	O'Hara	1.94	-70.752	166.634	15	-78.25	130.8	219.1
69	Chapman	2.49	-70.660	166.388	15	-12.51	6.37	0.76
70	-	1.28	-70.595	166.773	15	3.1	16.95	-7.55
71	Kirkby	3.18	-70.626	166.057	15	-21.05	119.26	65.53
72	McMahon	2.07	-70.668	165.783	15			-21.14
73	-	2.26	-70.583	165.594	15	-4.81	42.64	0.88
74	Zykov	8.22	-70.545	164.735	15	-23.75	7.73	-49.92
75	-	5.78	-70.517	164.512	15		32.86	-6.1
76	Lillie	9.86	-70.559	163.974	15	-260.88	232.93	159.71
77	Astakhov	5.28	-70.622	163.293	15	-64.99	15.1	20.62
78	-	2.9	-70.492	162.967	15	-6.56	20.03	33.76
79	Barber	3.82	-70.360	162.754	15	-21.44	-25.22	239.1
80	Gannutz	4.56	-70.307	162.161	15	-77.93	80.25	75.04
81	Rennick	27.78	-70.390	161.512	15	110.15	150.25	170.8
82	-	5.89	-70.205	160.911	15	-11.21	-8.56	50.68
83	Prior	5.75	-70.030	160.548	15	-23.1	54.24	20.55
84	Suvorov	10.74	-69.871	160.230	15	-16.5	-73.03	22.43
85	Manna	10.71	-69.623	160.026	15	-3.91	10.46	-10.83
86	-	5.68	-69.486	159.695	15	-39.08	156.44	-329.64
87	-	5.97	-69.471	159.533	15	-25.37	99.74	57.89
88	Tomlin	7.34	-69.362	159.054	15	83.8	134.37	-94.53
89	Paternostro	3.01	-69.282	158.668	15	-32.27	28.77	-37.16
90	McLeod	3.9	-69.294	158.401	15	-28.06	21.34	67.55
91	-	3.12	-69.243	158.348	15	3.34	1.55	-7.13
92	Jmaes Forbes	2.47	-69.138	157.995	14	-13.27	-1.95	-0.81
93	Matus Evich	9.27	-69.241	157.371	14	-1019.54	-491.13	324.92
94	-	5.09	-69.105	156.887	14	-114.14	-14.7	106.82
95	-	17.51	-69.015	156.257	14			66.2
96	-	6.69	-69.002	156.056	14			284.47
97	-	6.4	-68.947	155.461	14	-27.61	23.48	-265.84
98	-	8.43	-68.618	154.735	14	-33.29	11.91	-66.54
99	-	4.53	-68.415	154.333	14	-27.96	-21.35	0.36
100	Cook ice shelf (	57.59	-68.446	152.605	14	678.94	713.14	810.1
101	Cook ice shelf (	35.04	-68.662	151.526	14	-707.32	127.8	-89.8
102	Williams	14.63	-68.358	149.564	14	-191.92	219.8	55.15
103	-	49.1	-68.343	148.143	14	-477.79	264.8	449.12
104	Ninns	40.54	-68.215	147.271	14	-1388.17	-772.53	834.75
105	-	10.89	-67.986	146.855	14	-29.34	241.45	-6.1
106	-	11.24	-67.872	146.532	14	15.24	39.97	3.36
107	-	9.91	-67.707	146.271	14		-66.91	213.47
108	AAE	12.95	-67.518	145.571	14		147.87	4.86
109	Mertz	35.01	-67.171	145.283	14		1054.2	-6913.69
110	-	6.44	-67.008	144.083	14	-12.05	30.29	-69.85
111	Zelee	4.9	-66.775	141.246	14	-9.88	-25.18	-81.81
112	-	5.92	-66.697	140.756	14	-13.95	8.79	16.27
113	-	1.91	-66.680	140.380	14	-53.68	31.33	19.37
114	Astrolabe	8.37	-66.634	140.029	14	-65.94	116.17	-160.08
115	Liotard	4.77	-66.557	139.539	14	-64.51	24.6	30.55
116	-	8.38	-66.494	138.213	14	14.67	-156.19	205.43
117	Marret	7.22	-66.350	137.714	14		50.71	-18.59
118	Commandant	4.9	-66.375	136.602	14	-1.24	-95.25	-26.96
119	-	10.75	-66.166	135.687	14	-163.73	113.48	49.54
120	-	18.04	-66.068	135.105	14	-89.82	68.23	20.64

121	Dibble	14.75	-66.020	134.642	14	-83.7	13.82	103.24
122	-	8.61	-66.139	132.708	14		46.28	44.9
123	Freeman	7.74	-66.128	132.414	14		19.43	-17.19
124	Harrison	11.27	-66.184	131.298	14		106.28	-87.56
125	-	6.82	-66.156	130.962	14		54.17	5.54
126	May	6.1	-66.118	130.618	14		-92.18	201.26
127	-	9.93	-66.160	130.513	14		118.46	350.9
128	-	6.38	-66.201	130.361	14		23.84	216.74
129	Morse	6.68	-66.297	130.074	13		63.28	-42.22
130	-	7.15	-66.678	129.612	13		-15.3	-163.95
131	-	9.18	-66.890	129.429	13		255.93	-178.3
132	Frost	15.46	-67.063	129.128	13		230.24	-208.53
133	-	16.13	-67.084	128.631	13		206.16	212.55
134	-	14.03	-67.063	128.060	13		223.72	-106.73
135	De Haven	11.59	-66.963	127.694	13		-265.78	-287.25
136	Holmes	49.69	-66.755	127.112	13		-29.26	479.18
137	-	17.91	-66.309	126.234	13		215.46	174.84
138	Thompson	7.32	-66.669	123.648	13		58.55	-40.53
139	-	6.44	-66.699	123.191	13		56.89	109.96
140	-	33.15	-66.689	121.894	13			-229.24
141	-	45.93	-66.965	118.702	13		-103.1	52.99
142	-	11.17	-66.978	117.891	13		-68.82	-152.21
143	-	27.62	-66.867	116.962	13	96.72	-137.25	3.93
144	Totten	45.41	-66.774	115.989	13	-406.82	216.97	-63.58
145	Elliot	6.8	-66.486	115.275	13	49.58	-27.69	-8.38
146	Williamson	5.29	-66.412	114.427	13	-155.97	21.77	35.85
147	Whittle	9.05	-66.298	114.353	13	-90	-21.04	77.28
148	Fox	6.31	-66.178	114.400	13	-16.84	137.63	-176.45
149	-	8.06	-66.038	114.131	13	-172.1	54.88	-107.29
150	-	7.25	-65.836	113.432	13		80.54	-24.21
151	-	8.36	-65.758	113.148	13		69.81	46.52
152	Vanderford	16.73	-66.507	110.467	13		359.73	-321.42
153	-	16.73	-66.666	109.909	13		34.9	-113.68
154	Adams	18.39	-66.762	109.744	13		-23.8	-95.85
155	ANZAC	6.91	-66.828	109.491	13		130.97	-90.33
156	-	4.07	-66.845	109.263	13			-4.81
157	Bond	6.7	-66.885	109.017	13		47.27	41.06
158	-	4.24	-66.909	108.858	13		59.68	-56.3
159	-	3.43	-66.694	108.322	13		56.29	-74.42
160	Underwood	9.52	-66.607	107.918	13	-45.23	24.02	-59.89
161	Hawkins	2.02	-66.479	107.476	13	-23.22	170.92	-133.74
162	Robinson	2.48	-66.438	107.256	13	-55.47	49.77	-66.81
163	Snedeker	5.62	-66.401	106.842	13	-134.24	27.39	-27.9
164	-	3.52	-66.394	106.718	13	-208.34	194.85	-336.47
165	-	4.03	-66.365	106.598	13	-36.34	18.42	-2.66
166	Du Beau	3.46	-66.339	106.353	13	-49.37	25.18	-107.65
167	-	7.08	-66.162	105.242	13	-4.15	36.18	-67.22
168	Scott	17.57	-65.903	99.811	12	382.6	-1591.4	641.79
169	Denamn	35.88	-66.045	99.044	12	-1611.7	1605.38	1759.21
170	Roscoe	23.75	-66.356	95.199	12	37.36	-70.05	15.46
171	Helen	9.23	-66.594	93.769	12			74.39
172	Annenkova	18.2	-66.599	92.522	12	-13.23	8.89	-15.86
173	Burton Island	1.92	-66.664	90.555	12	-428.13	-49.56	103.35
174	-	3.46	-66.767	90.350	12		206.41	-255.15
175	-	10.48	-66.862	89.453	12		12.87	-71.13
176		0.82	-66.601	90.824	12		-17.45	14.73
177		2.44	-66.629	90.724	12		15.75	30.65
178		3.3	-66.653	90.509	12	42.95	-31.1	69.65
179		5.6	-66.703	90.379	12	-32.41	139.77	-138.56
180		5.5	-66.713	90.191	12	-107.64	185.26	-53.75
181	Kovarvyj	1.9	-66.730	89.943	12	-59	11.34	-7.91
182	Rjadovoj	1.76	-66.746	89.777	12	-14.84	33.09	-38.55
183	Posadowsky	13.68	-66.770	89.414	12	-404.56	202.33	-174.2
184	Zybkij	3.62	-66.733	89.088	12	-172.85	15.87	-5.82

185	Philippi	23.6	-66.607	88.244	12		-4193.36	136.29
186		72.7	-66.471	87.368	12		-6151.85	471.53
187		39.8	-66.440	86.042	12	-947.41	330.03	243.83
188		43.8	-66.698	84.695	12	356.38	465.99	369.21
189		15.9	-67.091	83.887	12		-92.42	-318.2
190		49.8	-67.243	83.075	12	1169.53	-7976.48	702.83
191		23.21	-67.392	82.011	12	574.42	-1000.12	448.83
192		22.6	-67.564	81.584	12	265.31	53.44	-19.85
193		3.8	-67.852	80.657	12	-9.04	3.76	13.05
194	Sorsdal	6.3	-68.637	78.030	12	22.66	8.95	266.45
195	Browns	2.4	-68.873	77.941	12			20.63
196	Chaos	3.7	-68.930	77.932	12			-33.26
197		3.16	-69.035	77.719	12	46.41	226.35	-143.18
198	Ranvik	7.35	-69.083	77.494	12	50.29	17.55	-48.02
199		1.43	-69.151	77.256	12	51.28	-93.4	
200	Hovde	2.99	-69.193	76.924	12	33.54	-42.58	
201		3	-69.219	76.762	12	13.54	-38.22	
202		2.7	-69.262	76.676	12	-0.34	-31.34	24.85
203	Dalk	2.8	-69.356	76.456	12	-34.82	21.98	6.8
204	Shennong	1.8	-69.437	76.020	12	10.33	-41.76	16.33
205		2.78	-69.482	75.845	12	-6.42	64.57	27.25
206	Polarboken	12.4	-69.517	75.674	12	-143.84	-43.94	83.12
207	Polar Record	13.18	-69.690	75.332	12	-1067.31	920.6	538.67
208	Polarforsching	7.78	-69.781	75.106	12	-87.26	182.49	-143.95
209	Polo	4.41	-69.762	74.840	12	-59.33	-88.7	-18.86
210	Polar Times	7.36	-69.760	74.599	12	420.96	863.46	-1040.55
211		3.57	-69.711	74.306	12			26.21
212	lambert	142.7	-69.031	72.271	8/9/11	791.97	1265.14	832.01
213		20.58	-68.558	69.992	8/9/11	17.82	-23.54	102.6
214		1.9	-67.737	66.809	8/9/11	-4.31	4.71	-6.9
215		1.9	-67.722	66.105	8/9/11	20.21	-27.11	-3.19
216		3	-67.644	65.444	8/9/11	12.85	-46.03	7.26
217	Strahan	1.7	-67.630	65.070	8/9/11		15.36	44.93
218		4	-67.559	64.602	8/9/11		-70.76	5.66
219		1.37	-67.503	64.187	8/9/11		3.84	7.31
220		2.9	-67.533	62.925	8/9/11		-9.58	5.5
221		1.8	-67.557	62.851	8/9/11		-23.82	22.2
222		1.7	-67.587	62.765	8/9/11		51.5	-40.72
223		1.15	-67.588	62.598	8/9/11		37.58	-27.39
224	Forbes	2.5	-67.579	62.552	8/9/11		-85.14	12.57
225		1.47	-67.553	62.359	8/9/11		17.19	26.3
226		0.56	-67.534	62.309	8/9/11		26.16	23.27
227	Utstikkar	3.8	-67.508	62.165	8/9/11		70.49	195.92
228	Jelbart	3	-67.482	61.290	8/9/11		-447.07	15.79
229	Taylor	2.6	-67.468	61.190	8/9/11		-47.71	50.37
230	Scoble	9.6	-67.378	60.849	8/9/11		-14.67	27.8
231		1.8	-67.353	60.510	8/9/11		13.13	33.26
232		2	-67.343	60.101	8/9/11			-111.71
233	Mulebreen	5.2	-67.385	59.593	8/9/11	-78.72	394.71	81.02
234	Cosgrove	3.9	-67.357	59.270	8/9/11	19.03	228.09	117.13
235		5.2	-67.381	59.164	8/9/11	-133.14	57.2	107.47
236	Hoseason	5.4	-67.241	58.827	8/9/11	-28.4	58.06	-105.29
237		1.3	-67.033	58.200	8/9/11	2.32	31.17	-63.73
238		5.5	-67.039	58.021	8/9/11			
239	Robert	11.7	-66.961	57.166	8/9/11	-95.67	152.65	209.17
240	wilson	4.7	-66.967	56.580	8/9/11	21.99	3.4	13.75
241	Seaton	6.2	-66.730	56.471	8/9/11	15.86	-208.89	173.69
242		1.5	-66.632	56.467	8/9/11	-44.58	-43.88	-15.41
243		0.65	-66.633	56.875	8/9/11	-22.6	19.87	42.8
244		2.28	-66.631	56.925	8/9/11	-38.67	23.63	-34.07
245		2.3	-66.578	57.185	8/9/11	-41.83	-7.15	-12.41
246		3.79	-66.342	56.365	8/9/11	-1.37	31.05	-39.9
247		5.3	-66.263	56.189	8/9/11	74.87	124.13	-50.34
248		1.9	-66.066	55.739	7		-20.9	59.69

249		0.75	-66.118	51.397	7		-11.89	27.41
250		2.6	-66.710	50.529	7		-8.83	-68.29
251		3	-66.794	50.632	7		-17.48	0.1
252	beaver	6.1	-66.957	50.595	7		1.53	-52.04
253		2.3	-67.068	50.992	7		13.52	77.98
254		1.3	-67.109	50.867	7			33.15
255	Auster	3.3	-67.133	50.704	7		73.74	111.03
256		0.77	-67.143	50.474	7		57.66	-11.5
257		2.2	-67.106	50.314	7		37.13	12.58
258		4.65	-67.101	49.940	7		60.31	12.73
259		1.5	-66.969	49.704	7		45.09	23.12
260		7	-66.933	49.352	7		-44.8	29.71
261		4.98	-67.215	48.784	7		34.41	-143.07
262		1.8	-67.261	49.228	7		-1.81	36.06
263		1.3	-67.318	49.113	7		41.81	4.87
264	Thyer	4.8	-67.498	48.521	7		8.61	2.6
265	Rayner	10.9	-67.560	48.315	7	62.9	259.56	184.44
266	Kichebside	3.8	-67.584	47.775	7	-32.47	34.23	166.1
267		2.33	-67.499	47.540	7	-30.92	8.28	19.95
268	Molle	10.1	-67.410	47.413	7	-148.98	77.12	2.89
269		1.9	-67.262	46.939	7	-36.34	-7.85	25.76
270		1.46	-67.222	46.646	7	-25.01	24.95	13.3
271		0.97	-67.415	46.328	7	9.77	-27.5	1.62
272	assender	3.49	-67.523	46.407	7	15.75	-11.05	23.53
273	hayes	4.39	-67.582	46.325	7	-17.5	33.46	162.27
274	Campbell	3.15	-67.702	45.672	7	-162.69	48.07	17.7
275		0.88	-67.655	45.248	7	-4.42	-18.36	9.03
276		3.2	-67.659	45.119	7	-21.91	-17.44	54.21
277	Shinnan	4.96	-67.829	44.572	7	-44.55	-5.99	-98.02
278		1.92	-67.905	44.474	7	-24.66	45.5	2.94
279	Rakuda	2.33	-67.946	43.887	7	-145.31	-7.25	9.52
280		1.5	-67.969	43.841	7	-21.65	-28.39	76.61
281		1.9	-67.989	43.779	7	-90.6	-17.93	49.11
282	Chijire	2.5	-67.991	43.393	7	-8.78	-0.04	28.52
283		1.43	-68.003	43.106	7	-49.23	-29.87	64.58
284	Akebono	3.2	-68.048	42.868	7	-98.37	43.34	-73.83
285		2.78	-68.227	42.459	7	-11.16	48.21	-20.77
286	Kasumi	2.95	-68.277	42.426	7	-154.98	190.42	48.66
287		2.88	-68.305	42.363	7	-82.34	78.05	41.18
288	Ichime	5.53	-68.351	42.171	7	11.17	-9.27	43.49
289	Langknattbreen	5.67	-68.416	41.608	7	-100.81	50.02	108.73
290	Darumabreen	5.48	-68.481	41.336	7	-96.34	-17.61	83.83
291	Omega	2.89	-68.574	41.070	7	-8.55	-15.82	213.74
292		2.17	-68.592	41.010	7	-38.03	-0.06	161.99
293		1.7	-68.615	40.946	7	-190.66	17.98	161.55
294	Oku-iwa	2.47	-68.659	40.737	7	-17.86	24.08	198.22
295	Tama	2.33	-68.709	40.359	7	-53.83	43.02	-7.99
296		1.9	-68.784	39.874	7			-9.5
297		2.58	-68.767	39.991	7			-15.74
298	Hazuki	1.1	-69.068	39.767	7	-30.59	12.85	17.54
299	Langhovde	2.57	-69.136	39.796	7	-49.01	28.12	23.29
300	Honnor	1.89	-69.327	39.878	7	-20.97	28.61	87.76
301	Telen	2.25	-69.569	39.722	7	-295.88	-6.94	237.34
302	Skallebreen	3.95	-69.592	39.570	7	-142.51	171.66	225.26
303		5.4	-69.831	39.184	7	-564.73	91.46	283.16
304	Shirase	8.95	-69.917	38.625	7	-3914.11	957.69	2357.97
305		7.77	-69.942	38.275	7	150.77	34.33	149.61
306		8.34	-69.779	38.176	7	-112.99	423.14	-158.52
307		11.1	-69.596	37.970	7	-267.72	166.55	93
308	kaya	13.6	-69.459	37.408	7	-797.38	779.5	551.69
309		45.3	-69.280	35.975	7	436.2	690.44	601.12
310		26.2	-68.886	34.314	7		-627.37	
311	stanjikovicha	46.7	-69.637	29.964	6/5		185.54	163.04
312		39.5	-70.432	25.052	6/5			195.54

313		39.5	-70.368	22.686	6/5			61.46
314	Beregovogo	75.4	-70.141	20.570	6/5	382.25	496.71	252.12
315	Entuziasty	44.4	-69.990	18.166	6/5		520.41	-917.17
316		47.7	-69.728	14.785	6/5		312.34	215.72
317		12.5	-70.095	12.132	6/5	-218.86	59.53	24.54
318		12.7	-70.116	11.237	6/5	42.88	159.67	64.45
319		15.5	-70.080	10.385	6/5	73.38	137.16	82.16
320		25.9	-70.193	8.388	6/5	-260.62	301.03	-777.27
321		21.8	-70.169	6.950	6/5	-349.68	157.77	112.22
322		16.6	-70.160	5.927	6/5	-66.05	118.53	76.26
323		28.4	-70.273	5.221	6/5	35.12	-191.6	44.58
324		29.7	-70.252	3.490	6/5	-82.61	32.85	32.33
325		28.9	-70.207	1.835	6/5	-101.05	-45.39	33.52
326		16.8	-70.120	0.659	6/5	321.05	-194.83	-487.81
327	Bellinsgauzena	50	-69.949	-0.626	6/5	656.34	895.1	587.45
328		19.8	-70.351	-2.174	6/5	-167.07	-423.49	-18.55
329		34.6	-70.557	-4.447	6/5	251.69	227.75	351.69
330		44.4	-70.833	-8.793	6/5	-912.1	225.51	120
331		26	-71.166	-10.804	4	41.24	-130.06	49.94
332		24	-71.992	-11.767	4	-66.01	-8.17	-39.67
333		60.2	-72.293	-13.944	4	-836.16	164.61	185.78
334		30.9	-72.453	-15.571	4	-734.03	148.86	143.28
335		25.7	-72.639	-16.932	4	101.24	179.03	147.01
336	Veststraumen	34.8	-72.701	-18.641	4	-170.84	105.53	36.44
337	Stancomb-willa	61.2	-74.532	-23.529	4		1154.25	1185.71
338		49.1	-75.641	-26.166	4	300.33	499.85	324.4
339		4.8	-76.097	-26.702	4	-410.51	-63.98	235.3
340		4	-76.220	-27.757	4	44.96	-20.12	-29.9
341		1.9	-76.461	-29.444	4	-20.45	16	-15.88
342		2.5	-76.526	-29.557	4	-6.7	-6.87	-3.54
343		3.3	-76.750	-30.464	4	-45.89	54.53	-32.12
344		1.2	-76.813	-30.671	4	2.62	-10.16	6.33
345		4.5	-77.133	-31.892	4	-26.83	-16.37	62.17
346		3.7	-77.255	-33.347	4	108.68	87.86	-139.15
347		3.2	-77.379	-33.884	4	-27.37	-11.2	-6.55
348	Schweitzer	6.7	-77.756	-34.878	4	140.79	263.39	141.81
349		8	-78.109	-35.556	4		171.39	-317.71
350	Filchner	139.5	-78.517	-40.066	4	-3792.18	1627.15	862.94
351	Ronne	407.8	-76.309	-57.933	4		-2409.21	1033.17

[1]Arbitary glacier number used in this study

[2]Glacier name, where known, obtained from the Scientific Committee on Antarctic Research (SCAR) Composite Gazetteer of Antarctica (https://data.aad.gov.au/aadc/gaz/scar/)

[3]Measured glacier width (km)

[4]Glacier terminus position changes between two time-steps measured using area changes within a reference box, divided by glacier width (see Materials and Methods)
